# Pneumothorax Ex-vacuo or Trapped Lungs Appearing as Iatrogenic Hydropneumothorax: A Case Report and Review of Non-expandable Lungs (NEL)

**DOI:** 10.7759/cureus.41814

**Published:** 2023-07-13

**Authors:** Saquib Siddiqui, Umair Falak

**Affiliations:** 1 Respiratory Medicine, Queen Elizabeth Hospital Gateshead, Newcastle Upon Tyne, GBR

**Keywords:** pneumothorax ex vacuo, thoracentesis, non expandable lungs, trapped lungs, hydropneumothorax, pneumothorax

## Abstract

Non-expandable lungs are usually diagnosed after a pleural intervention. It can be challenging to differentiate between an iatrogenic pneumothorax and a new diagnosis of non-expandable lungs following a pleural intervention. The correct assessment can save the patient from undergoing the insertion of an unnecessary intercostal chest drain, which often leads to catastrophe. Suspicion and early evaluation remain the keys, particularly in patients with chronic effusion. Often the diagnosis is reached through a combination of history, pleural fluid analysis, and radiological features such as the absence of a straight line in the chest X-ray, which is commonly found in a true hydropneumothorax, along with computed tomographic evidence of chronic effusion with thick pleural rind. Although not routinely performed, pleural manometry can confirm the diagnosis of trapped lungs.

We present our case, where a 64-year-old woman with metastatic oesophageal cancer developed a right-sided effusion. The post-procedure chest X-ray following therapeutic aspiration of the pleural fluid gave an impression of iatrogenic hydropneumothorax, which on further careful assessment revealed a rather pneumothorax ex-vacuo along with effusion due to underlying trapped lungs. We present a review of non-expandable lungs.

## Introduction

Non-expandable lungs are not an uncommon finding in respiratory medicine. It is usually diagnosed after a pleural intervention. It can be a challenge to differentiate between an iatrogenic pneumothorax and a new diagnosis of non-expandable lungs following a pleural intervention. In the setup of our services, this means that the acute medical team, especially the on-call team, will come across this and will need to make decisions about intervention along with our respiratory colleagues. Here we present our case (development of pneumothorax ex-vacuo along with effusion post-pleural fluid aspiration due to underlying trapped lungs) with a review of the topic.

## Case presentation

**Figure 1:**
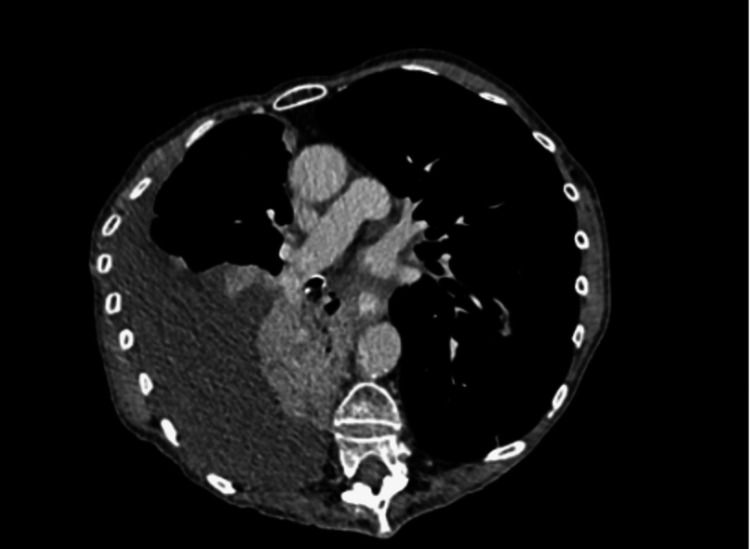
A CT scan of the chest shows a right-sided pleural effusion with a collapse of the right lower lobe.

**Figure 2:**
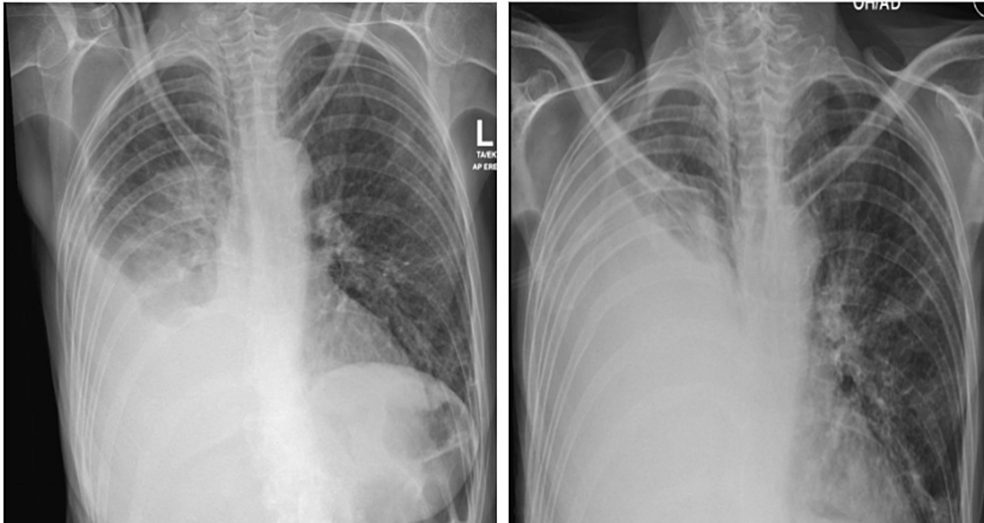
The two chest X-rays performed within a gap of three days reveal worsening right-sided pleural effusion.

A 64-year-old woman with a diagnosis of metastatic oesophageal cancer was admitted to the palliative care unit. She had metastatic disease of the bones and lungs. There was also a locally advanced invasion leading to obstruction of the right lower lobe. Metastatic deposits in the spine and right femur were causing considerable pain. She had undergone radiotherapy for them, but the pain remained uncontrolled. She was also suffering from dysphagia due to oesophageal narrowing. Her body mass index (BMI) was only 18 kg/m2. She had significant pitting oedema to the knees bilaterally, which was thought to be secondary to low albumin levels. She was also suffering from anxiety and panic attacks. Her pain control was optimised, and she was started on a syringe driver. The dietary team started supplements and advised on special diets. She developed a new oxygen requirement. A pulmonary embolism was ruled out by a computed tomography pulmonary angiography (CTPA) scan. Radiological investigations confirmed a worsening right pleural effusion (Figures 1-2).

**Figure 3:**
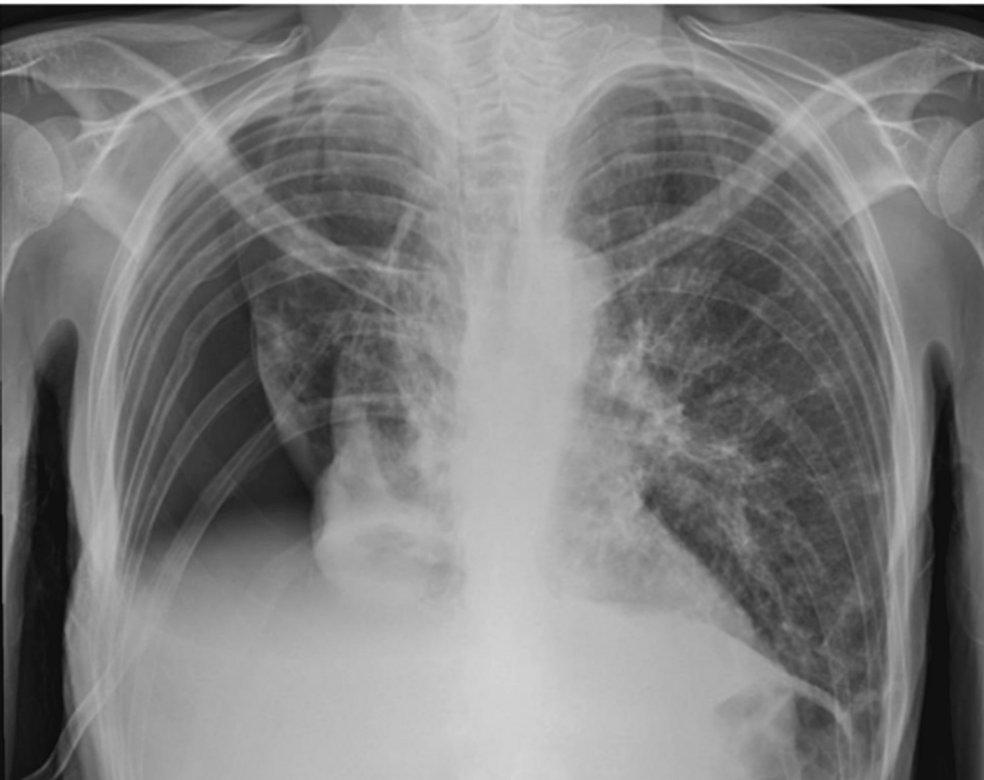
Post-right thoracentesis reveals an appearance suggestive of right-sided iatrogenic hydropneumothorax camouflaging the right trapped lung, which leads to the development of pneumothorax ex-vacuo (No typical straight line for a typical hydropneumothorax).

The respiratory team was consulted for possible therapeutic pleural fluid aspiration to provide symptomatic relief and possibly aid in the weaning of supplementary oxygen. She had never had any pleural intervention done for the fluid before. Bedside thoracic ultrasound after discussion with the patient revealed a simple right-sided effusion. Therapeutic pleural fluid aspiration was done under ultrasound guidance. The patient noted an immediate benefit post-procedure, and her oxygen requirement started to decline. However, the post-procedure chest X-ray showed significant hydropneumothorax (Figure 3).

The patient was reviewed again; she was still feeling much better, and her oxygen requirement had continued to go down. She denied feeling pain in her chest. The trachea was central, with reduced breath sounds on the right side of the chest. A closer look at the X-ray showed that there was no straight line that we would assume to see with a typical hydropneumothorax. Another thing to note is that the lungs had a similar appearance before drainage. The impression based on the patient’s clinical stability and the hints on the X-ray was that this was a trapped lung causing pneumothorax ex-vacuo following aspiration, which gives the appearance of a hydropneumothorax. We chose to monitor the patient, and she continued to remain stable with an improvement in her breathing. As she had trapped lungs, further therapeutic pleural aspirations would be at risk. An intrapleural catheter was judged not to be in her best interest based on her advanced cancer and frailty. This was discussed with the patient, and a shared decision was reached. She was fast-tracked and discharged home with community palliative team support. She passed away peacefully after a few weeks.

## Discussion

The two pleural entities for non-expandable lungs (NEL) are lung entrapment and trapped lungs. Lung entrapment is an active process that occurs due to persistent active inflammation, resulting in effusion with pleural restriction (infection, inflammation, or malignancy). Trapped lungs, on the other hand, are a remote process resulting from old or chronic inflammation that results in the development of fibrosis during the healing process in the pleural surface (post-surgery, haemothorax, pneumothorax, infection, pleuritis, chronic effusion due to any cause). It is often defined as a lung that is unable to fill the thoracic cavity due to the development of fibrous restrictive pleural surfaces (visceral), which prevent complete expansion. It was first described by Moore in 1967 [1]. In those days, trapped lungs were found following the development of therapeutic pneumothorax while treating tuberculosis. Current studies show an incidence rate of 20% in patients who undergo therapeutic thoracentesis, whereas 30% of patients with malignant pleural effusion will eventually be found to have trapped lungs following effusion drainage [2]. Trapped lungs are more frequent in malignant effusions as the malignant tissue encases the visceral layer of the pleura, resulting in incomplete re-expansion of the lungs post-drainage. The fibrinolysis peel also restricts the expansion of lung parenchyma, resulting in the development of high negative intrapleural pressure, which eventually leads to increased fluid formation and the persistence of the pleural effusion [2]. This high negative pressure can also lead to air leaks, resulting in the appearance of a hydropneumothorax. This specific form of pneumothorax resulting from a pressure difference due to trapped lungs is known as pneumothorax ex-vacuo. The term pneumothorax ex-vacuo was originally coined to describe the condition seen in bronchial obstruction. As a result of bronchial obstruction, a part of the lung collapses and fails to re-expand, leading to the formation of a vacuum that is filled with air. As mentioned earlier, we can see a similar situation in non-expandable lungs after pleural intervention. It can happen due to the presence of a bronchopleural or alveolar-pleural fistula, which creates a unidirectional airflow into the pleural cavity; this is referred to as a pressure-independent mechanism [3,4]. Another possible mechanism is called pressure-dependent, where once the fluid is removed, the lungs do not re-expand to fill this space, and a vacuum is created [4, 5]. The negative pressure created causes air to leak in to fill the vacuum [4,5]. The source of the air is uncertain, but a possible theory is that small defects form on the visceral pleura due to the excessive negative pressure created. Through these defects, air leaks into the vacuum until the negative pressure settles. Another possible source is the entrance of atmospheric air through the needle's tract. This type of pneumothorax is a negative pressure event and will not enlarge with time, staying the same shape and size until it is replaced with fluid again. Therefore, performing serial chest X-rays might be helpful to monitor progress in cases of doubt, thus avoiding the insertion of an unnecessary chest drain, which will lead to a worsening of the underlying mechanism by creating a continuous source of negative pressure leading to non-healing. The survival rate in patients with pneumothorax ex-vacuo treated conservatively is better than in those who underwent chest drain insertion [6].

Non-expandable lungs (NEL) can be caused by a problem in the airways, lung tissue, or pleura [4,7]. A bronchial obstruction might lead to a lung collapse, which will fail to re-expand. Any chronic process in the lung tissue that affects the elasticity of the lungs, for example, pulmonary fibrosis, can rarely cause a similar situation. In essence, lung entrapment is an active process that is ongoing, leading to the visceral pleura being coated and preventing it from re-expanding [4]. Lung trapping, on the other hand, is due to previous insults that have led to the development of chronic effusion. Almost any effusion, including parapneumonic effusions, post-cardiac surgery, haemothorax, inflammatory pleuritis, chest radiation, and malignant pleural effusions, can lead to lung trapping [8]. Lung trapping has even been described due to hepatic hydrothorax [8]. They can be differentiated by pleural manometry. Pleural space elastance (change in pleural pressure or amount of pleural fluid removed) of more than 14.5 cm of H2O/L will be diagnostic for trapped lungs [4,5]. Although it is the gold standard diagnostic test, it is rarely used as it is time-consuming and, due to its technical difficulties, requires additional training. If performed, it could be a useful tool to predict successful pleurodesis and the early identification of non-expandible lungs.

Most patients with pneumothorax ex-vacuo will be asymptomatic. On a chest X-ray, certain subtle hints can be seen that help differentiate a pneumothorax ex-vacuo from a true pneumothorax. Usually, the lung tissue keeps the same contour and shape as before the intervention, and the pneumothorax is usually basilar [4]. There might be no clear straight line, as seen in a hydropneumothorax associated with ipsilateral mediastinal shift and volume loss [4]. The nature of pneumothorax ex-vacuo is such that it does not expand or worsen further and is rapidly replaced by fluid, as mentioned, which can be demonstrated on serial X-rays [4]. A contrast computed tomography (CT) scan of the chest might show underlying secondary causes of non-expandable lungs and lung trapping.

As pneumothorax ex-vacuo is a formidable problem, it is crucial to see if we can predict it before the procedure itself. Certain subtle hints might be seen in radiology. A CT scan of the chest showing pleural thickening and bronchial obstruction might predict non-expandable lungs. Trapped lungs can also be confirmed with air-contrast computed tomography, which reveals thick visceral pleura. The thick pleural rind can also be visualised directly during video-assisted thoracoscopy (VATS). Bedside ultrasound has become a more common and popular tool for examining the chest nowadays. Certain features have been described that may hint at a non-expandable lung. On m-mode in usual pleural effusion, the lung can be seen making a sinusoidal pattern; this has been noted to be lost in non-expandable lungs, known as the "absent sinusoidal sign" [9]. Along with the absent sinusoidal sign, other common bedside ultrasound findings are usually pleural thickening, pleural nodularity, and loculated effusion. Advanced pleural ultrasound findings in such cases might show less movement and deformation in trapped lungs compared to expandable lungs [10].

The pleural fluid study is usually lymphocytic exudate in lung entrapment due to active inflammation and becomes more of a transudate or protein discordant, paucicellular, in trapped lungs [11, 12].

Most patients remain asymptomatic or experience minimal exertional dyspnoea; therefore, simple observation is sufficient without intervening. Conservative management remains the treatment of choice in asymptomatic patients, as intervening with an intercostal chest drain does not seem to improve clinical benefit [6]. Any placement of intercostal drains increases the risk of further injury through persistent air leakage. Surgical decortication can be performed for extremely symptomatic patients with trapped lungs [11,13]. Treatment of the underlying cause remains the most important intervention.

## Conclusions

It is essential to have an intuition for a possible underlying trapped lung, particularly in patients presenting with chronic effusion. Therefore, if needed to perform any pleural intervention for symptomatic relief, the post-procedure chest X-ray appearance of hydropneumothorax or pneumothorax should be assessed carefully to avoid the unnecessary insertion of a chest drain, which can result in more harm by creating a persistent air leak. Simple observation without intervention is the key to managing asymptomatic patients. Invariably, such patients are stable and remain asymptomatic despite their radiological appearance. Our message here is clear: think about why the lungs have not expanded despite fluid aspiration and avoid inserting chest drain tubes in patients with trapped lungs.
